# Subcellular determinants of orthoflavivirus protease activity

**DOI:** 10.1016/j.jbc.2025.110451

**Published:** 2025-07-05

**Authors:** Lochlain Corliss, Chad M. Petit, Nicholas J. Lennemann

**Affiliations:** 1 Department of Microbiology, University of Alabama at Birmingham, Birmingham, Alabama, USA; 2 Department of Biochemistry and Molecular Genetics, University of Alabama at Birmingham, Birmingham, Alabama, USA

**Keywords:** orthoflavivirus, protease, polyprotein, positive-sense RNA virus, live-cell imaging, flavivirus, endoplasmic reticulum, molecular tools

## Abstract

Orthoflaviviruses are small, enveloped, positive-sense RNA viruses that cause over 500 million infections globally each year for which there are no antiviral treatments. The viral protease is an attractive target for therapeutics due to its critical functions throughout infection. Many studies have reported on the structure, function, and importance of orthoflavivirus proteases; however, the molecular determinants for cleavage of intracellular substrates by orthoflavivirus proteases and how these factors affect viral fitness are unknown. In this study, we used our fluorescent, protease-activity reporter system to investigate the subcellular determinants involved in orthoflavivirus protease cleavage. By modifying our reporter platform, we identified endoplasmic reticulum (ER) subdomain localization and membrane proximity of the substrate cleavage site as two previously uncharacterized molecular determinants for cleavage. We also altered the amino acid composition of the reporter recognition motif to introduce sequences present at the cytoplasmic cleavage junctions within orthoflavivirus polyproteins and found that each protease processed the sequence located at the junction between NS4A and the 2K peptide least efficiently. Live-cell imaging revealed that cleavage of the NS4A|2K motif is significantly delayed compared to the capsid cleavage sequence. We further determined that introducing a more efficient cleavage sequence into the NS4A|2K junctions of orthoflavivirus infectious clones abolished virus recovery. Overall, this study identifies ER subdomain localization and membrane proximity of the recognition motif as molecular determinants for cleavage by orthoflavivirus proteases and provides insight into the role that sequence specificity plays in the coordinated processing of the viral polyprotein and establishing productive infections.

Orthoflaviviruses belong to a large family of viruses with positive-sense, single-stranded RNA genomes. The viruses in this genus are arthropod-borne and predominantly transmitted by several mosquito species, primarily of the *Aedes* and *Culex* genera (1, 2, 3, 4). The arthropod vector of orthoflaviviruses is the main contributor to sporadic epidemic outbreaks in warm geographic locations with high mosquito populations, such as Africa, South and Central America, and Asia (5, 6). However, because of persistent global warming and mosquito migration, previously naive regions, like the highly populated Gulf Coast and Southwestern coastal region of the United States, are becoming habitable locations for these viral vectors and are at risk of becoming hotspots for outbreaks (7, 8). Members of this viral family pose a significant global health concern with dengue virus (DENV) causing an overwhelming ∼ 390 million cases per year (9, 10). Infection with orthoflaviviruses can cause disease leading to severe clinical manifestations in multiple organ systems of the human body. Disease symptoms can include hemorrhagic fever or shock, caused by DENV and yellow fever virus (YFV), encephalitis, caused by West Nile virus (WNV), and birth defects such as microcephaly as a result of Zika virus (ZIKV) infection in pregnant women (11, 12, 13). Orthoflavivirus epidemics have devastating effects on human health as well as significant social and economic burdens to the affected regions (14, 15, 16). There are few safe and effective vaccines and no approved antiviral treatments for diseases caused by orthoflaviviruses (17, 18, 19). Given the worldwide prevalence of orthoflavivirus infections, it is crucial to gain a more in-depth understanding of the molecular processes of these pathogens to aid in the development of effective preventions and treatments.

Upon viral entry and uncoating, the orthoflavivirus genome is translated at the host cell endoplasmic reticulum (ER) into a single polyprotein that is associated with the ER through multiple transmembrane domains (20, 21). The polyprotein is then processed by viral and host proteases into 10 functional subunits, consisting of three structural proteins (capsid, pre-membrane, envelope) and seven non-structural (NS) proteins (NS1, NS2A, NS2B, NS3, NS4A, NS4B, and NS5) (22, 23). The viral serine protease, a complex between NS2B and NS3 (NS2B3), facilitates all the cytoplasmic cleavage events of the polyprotein (22, 24, 25). NS3 is a soluble, cytoplasmic protein consisting of a protease and helicase domain; however, it remains localized at the ER through its association with NS2B (26). NS2B has multiple transmembrane domains that anchor it to the ER while NS3 associates with the cytoplasmic loop of NS2B. This hydrophilic domain of NS2B is known to serve as a conserved cofactor that is required for the catalytic activity of the protease (27, 28, 29). Previous studies have identified a conserved substrate recognition motif based on the sequences processed within the orthoflavivirus polyprotein, and *in vitro* biochemical assays with a truncated recombinant DENV protease have shown this enzyme cleaves a broad range of sequences within the substrate, which contrasts with the WNV protease (25, 30, 31). However, there are limited studies investigating the intracellular activity of various NS2B3 protein complexes and how this contributes to infection.

Given the prominent role of the virally encoded protease in the virus lifecycle and disease pathogenesis, it has long been an attractive target for viral inhibitors (32, 33, 34). To date, all antiviral therapeutics developed against the orthoflavivirus protease that show promising pre-clinical efficacy have been unsuccessful in clinical testing (35). Many inhibitors have been found to only be effective in biochemical assays, suggesting a gap in knowledge of the complex molecular activities of this viral protein in infected cells (36). Thus, a deeper understanding of the mechanism of action behind NS2B3-mediated cleavage during infection is vital for the design and development of effective antivirals targeting this essential viral protease complex.

## Results

### Intracellular reporter system for assessing substrate cleavage efficiency by orthoflavivirus proteases

**Figure 1:**
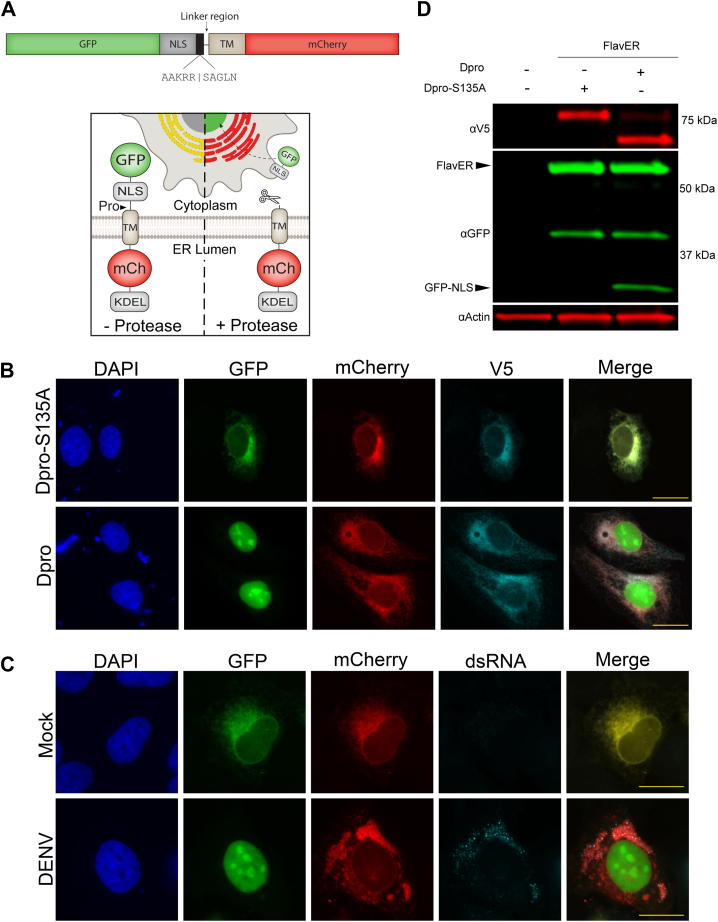
**Design and validation of flavivirus protease activity reporter (FlavER).***A*, linear and cartoon model of FlavER. (*A*, *top panel*) Linear model of FlavER. The WT FlavER reporter consists of a GFP fused to an NLS followed by a consensus viral protease cleavage motif that is fused to a TM domain followed by an mCherry fluorescent protein. *Black* box represents the sequence cleaved by the viral protease and *black* line represents the linker region between the cleavage motif and the TM. (*A*, *bottom panel*) cartoon model of FlavER function. In cells lacking viral protease expression (*left*), the GFP and mCherry signals co-localize at the ER. In cells expressing a flavivirus protease (*right*), the NS2B/NS3pro recognition sequence is cleaved, allowing for nuclear translocation of the reporter signal. *B*, immunofluorescence (IF) microscopy of FlavER-expressing U2OS cells transfected with WT DENV protease (Dpro) or catalytically inactive DENV protease (Dpro-S135A). GFP signal is shown in *green*, mCherry is shown in *red*, V5 staining is shown in *cyan*, and nuclei are stained with DAPI shown in *blue*. Scale bars represent 20 um. *C*, IF microscopy of FlavER-expressing U2OS cells infected with DENV at an MOI of 10. GFP signal is shown in *green*, mCherry is shown in *red*, dsRNA staining is shown in *cyan*, and nuclei are stained with DAPI and shown in *blue*. Scale bars represent 20 um. *D*, immunoblot for V5, GFP, and actin of U2OS cells co-transfected with FlavER and the indicated DENV protease. Labeled arrows denote the full-length (FlavER) and cleaved (GFP-NLS) reporter construct. Dpro-S135A represents the catalytically inactive DENV protease. ∗, denotes an intermediate product caused by non-specific proteolysis of mCherry as previously reported (37, 99).

To study orthoflavivirus protease specificity for intracellular substrates, we utilized our recently developed protease-dependent reporter system (37). This modified reporter construct is designed with a green fluorescent protein (GFP) fused to a nuclear localization signal (NLS) followed by an interchangeable 10 amino acid protease recognition motif. The recognition motif is then fused to a transferrin receptor transmembrane domain (TM) that anchors a red fluorescent protein (mCherry) into the lumen of the ER (Fig. 1*A*). In cells co-expressing our reporter with a catalytically inactive V5-tagged DENV protease (Dpro-S135A), both fluorescent signals stay co-localized at the ER; however, in cells co-expressing our reporter with an active V5-tagged DENV protease (Dpro), we observe nuclear translocation of the GFP signal indicating cleavage of the recognition motif while the mCherry signal remains localized to the ER (Fig. 1*B*). Similarly, we observe the same phenotype of nuclear translocation of GFP and ER-retention of mCherry in reporter-expressing cells that are infected with DENV (Fig. 1*C*). Probing the lysates of cells transfected under the same conditions as Figure 1*C* revealed a ∼30 kDa cleavage band (GFP-NLS) from cells expressing the active DENV protease, while this cleavage band was absent in cells expressing the inactive construct (Fig. 1*D*). The same cleavage fragment is observed from the lysates of reporter-expressing cells that are infected with multiple orthoflaviviruses (37). Together, these results indicate that reporter cleavage can be observed visually using immunofluorescent staining or immunoblotting methods.

### ER membrane proximity as a molecular determinant of DENV protease cleavage

**Figure 2:**
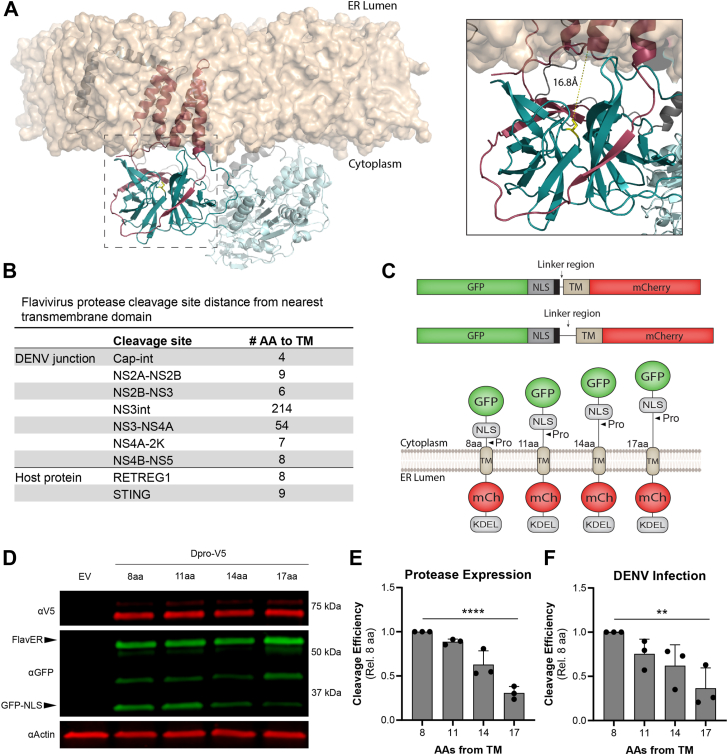
**Membrane proximity of substrate cleavage site as a molecular determinant for cleavage**. *A*, *Left*, molecular model of the NS2B3 complex in relation to a lipid bilayer. *Right*, zoom-in of the boxed region. NS2B is shown in *dark pink*, the NS3 protease domain is shown in *dark cyan*, the catalytic serine is shown in *yellow*, the NS3 helicase domain is shown in *light cyan*, NS4A is shown in *gray*, and the lipid bilayer is shown in *ta*n. Distance from the catalytic serine to the membrane is represented as a *yellow line* (16.8 Å). *B*, Table showing the predicted number of residues of the protease recognition site from the nearest TM domain within the cytoplasmic cleavage junctions of flavivirus polyproteins. *C*, linear and cartoon models representing the reporter constructs with lengthened flexible linker regions between the protease recognition site and the TM domain. *D*, immunoblot for V5, GFP, and actin of U2OS cells co-transfected with the indicated reporter construct and Dpro. Labeled arrows denote the full-length (FlavER) and cleaved (GFP-NLS) reporter construct. *E* and *F*, quantification of cleavage assays from reporter-expressing cells transfected with Dpro (*D*) or infected with DENV at an MOI of 3 (*E*). Data are presented as efficiency of cleavage determined by intensity ratios in AU of the cleaved to total reporter signal relative to viral protease expression. Quantification represented as cleavage efficiency relative to the FlavER-8aa construct. Each bar represents the average cleavage efficiency of three experiments per condition and *black circles* indicate the cleavage efficiency calculated for each replicate. Statistical significance was determined by unpaired t-tests. ∗∗*p* < 0.01, ∗∗∗∗*p* < 0.0001.

We sought to use our protease activity reporter system to assess the potential molecular determinants involved in cleavage specificity. During our validation of the FlavER reporter, we found that the construct must have a TM domain that anchors it to the ER membrane in order to be cleaved by the DENV protease, suggesting that membrane association of intracellular substrates is an important factor for protease specificity (37). These results led us to hypothesize that the proximity of the substrate cleavage site to the ER membrane may serve as a cleavage determinant. This hypothesis is supported by molecular modeling, which shows the catalytic serine of the ER-tethered protease domain is 16.8 Å from the membrane, suggesting that the protease is highly restricted to substrates proximal to the ER membrane (Fig. 2*A*). Consistently, the majority of cytoplasmic cleavage junctions within the DENV polyprotein are predicted to be positioned less than 10 amino acids away from the closest TM domain, suggesting an optimal membrane proximity for efficient substrate cleavage, as has been reported for other membrane-constrained proteases (Fig. 2*B*) (38). To determine whether the membrane proximity of the cleavage site is involved in cleavage efficiency, we employed the use of flexible linkers, which are often used in studies to separate functional protein domains and allow them to occupy positions at greater distances without affecting their functions (39, 40). We modified our WT FlavER to increase the number of amino acids by inserting glycine-glycine-serine flexible linkers downstream of P5 in the cleavage sequence of the reporter (Fig. 2*C*). These constructs were then used in intracellular cleavage assays where we co-expressed the DENV protease with a reporter encoding a membrane proximity of 8, 11, 14, or 17 residues and probed the cell lysates for GFP to reveal the ratio of full-length to cleaved reporter and the V5 epitope tag to determine the expression of the viral protease. These experiments resulted in a statistically significant decrease in reporter cleavage efficiency as the number of residues in the linker region increased from 8 to 17 residues (Fig. 2, *D* and *E*). These results were further recapitulated in the context of infection when cells expressing the membrane proximity reporters were infected with DENV for 24 h (Fig. 2*F*). These data suggest that membrane proximity of the protease recognition site is involved in the efficiency of substrate cleavage and that there is an inverse correlation between increased distance and cleavage efficiency.

### ER subdomain localization as a molecular determinant DENV protease cleavage

**Figure 3:**
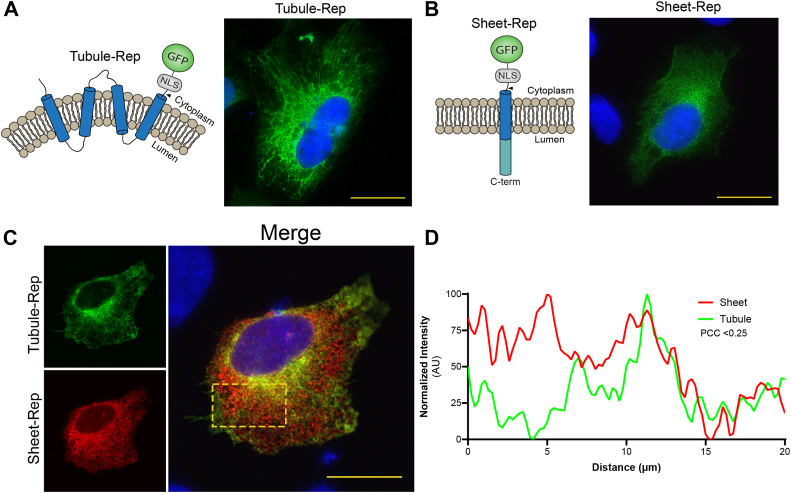
**Development of reporters localized to specific ER subdomains**. *A-B*, cartoon model and representative IF microscopy images of U2OS cells expressing GFP-Tubule-Rep (*A*) and GFP-Sheet-Rep (*B*). GFP signal is shown in *green*, and nuclei are stained with DAPI and shown in *blue*. Scale bars represent 20 um. *C*, IF microscopy of U2OS cells co-transfected with GFP-Tubule-Rep and mCh-Sheet-Rep. GFP-Tubule-Rep shown in *green*, mCh-Sheet-Rep shown in *red*, and nuclei are stained with DAPI and shown in *blue*. Scale bars represent 20 um. *D*, plot profile of normalized signal intensities from representative GFP-Tubule-Rep (*green*) and mCherry-Sheet-Rep (*red*) expressing cells.

**Figure 4:**
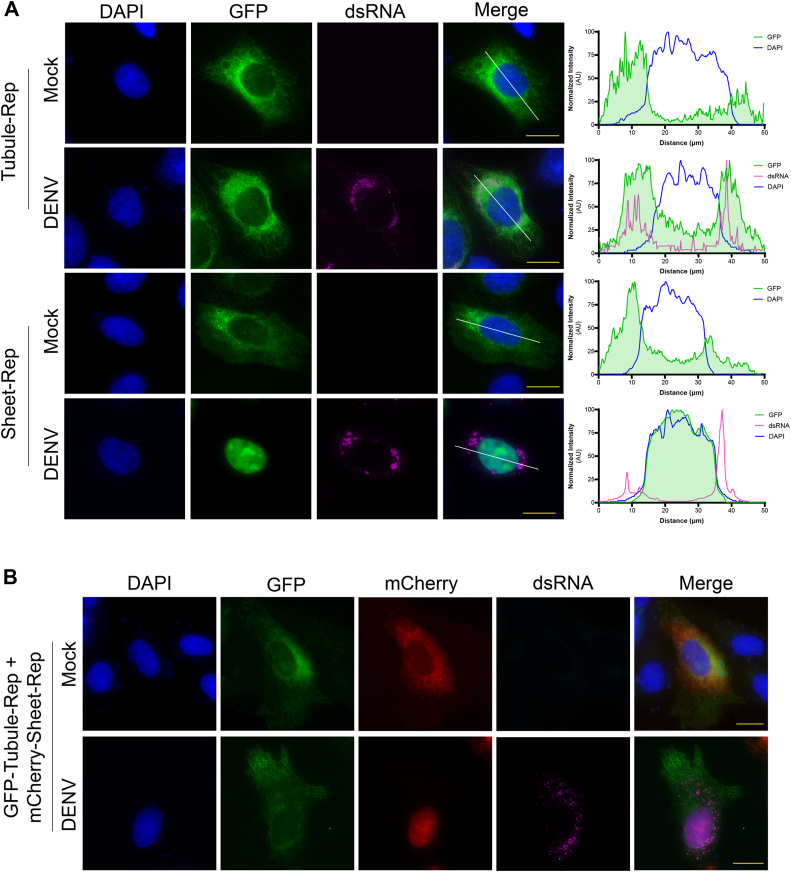
**Substrate localization to ER subdomains as a molecular determinant for cleavag**e. *A*, IF microscopy and plot profiles of mock or DENV-infected U2OS cells expressing GFP-Tubule-Rep (*top two panels*) or GFP-Sheet-Rep (*bottom two panels*). Reporter constructs are shown in *green*, dsRNA is shown in *magenta*, and nuclei are stained with DAPI and shown in *blue*. Scale bars represent 20 um. *B*, IF microscopy of mock (*top*) or DENV-infected (*bottom*) U2OS cells co-expressing GFP-Tubule-Rep and mCherry-Sheet-Rep. GFP-Tubule-Rep shown in *green*, mCherry-Sheet-Rep shown in *red*, dsRNA shown in *magenta*, and nuclei are stained with DAPI and shown in *blue*. Scale bars represent 20 μm.

Although the ER is a single, interconnected network composed of a contiguous membrane, this dynamic organelle is known to have functionally and structurally distinct subdomains consisting of curved tubules and flat sheets (41, 42, 43, 44, 45). Because the established relationship between the ER and orthoflavivirus replication cycle has been well documented, we next wanted to investigate the effects of substrate localization to specific ER subdomains on cleavage efficiency by the DENV protease (20, 46, 47, 48, 49, 50, 51, 52). To examine whether the DENV protease has a specificity for cleavage of substrates localized to ER tubules *versus* sheets, we modified our reporter system to fuse a fluorescent protein, NLS, and protease recognition motif to host proteins known to localize to these different subdomains of the ER. For the ER tubule construct (Tubule-Rep), our reporter motif (GFP-NLS-cleavage site) was fused to the C-terminus of reticulon-4a (RTN4A), while the sigma 1 receptor (SIGMAR1) was inserted downstream of the reporter motif for the ER sheet construct (Sheet-Rep) to ensure proper topology and membrane proximity of the cleavage site (Fig. 3, *A* and *B*). These two biochemically and functionally distinct host proteins have been shown to perform mutually exclusive activities in ER shaping, including promotion and maintenance of the separate subdomains in which they reside (53, 54, 55). Using fluorescence microscopy of cells expressing Tubule-Rep, we observed a web-like network of tubular structures extending from the nuclear envelope into the cell periphery (Fig. 3*A*). In contrast, we observed structures with a more broad and planar appearance consistent with ER cisternae morphology in cells expressing the Sheet-Rep construct (Fig. 3*B*). We then swapped the GFP of Sheet-Rep with mCherry to allow for distinguishable co-expression of the two constructs and visually observed that the respective fluorescent signals localized to the distinct subdomain to which they are targeted (Fig. 3*C*). Further plot profile analysis revealed that although both reporter constructs reside within the same organelle, there is weak co-localization between the two signals with a Pearson’s correlation coefficient of <0.25 (Fig. 3*D*). To determine the impact of ER subdomain localization on substrate cleavage by the DENV protease, Tubule-Rep and Sheet-Rep were individually transfected into cells that were either mock-infected or DENV-infected. While we observed a strong GFP signal in the nuclei of DENV-infected cells expressing Sheet-Rep, we observed no nuclear translocation of the GFP signal in infected Tubule-Rep-expressing cells, suggesting that the DENV protease does not efficiently cleave substrates specifically localized to ER tubules during infection (Fig. 4*A*). These findings were further supported in the context of co-expression, where nuclear translocation of the reporter signal was only observed with mCherry-Sheet-Rep in cells transfected with both reporter constructs (Fig. 4*B*). Together, these results indicate that the DENV protease has a strong preference for substrates localized to ER sheets.

### Orthoflavivirus proteases exhibit sequence-specific cleavage efficiency profiles

**Figure 5:**
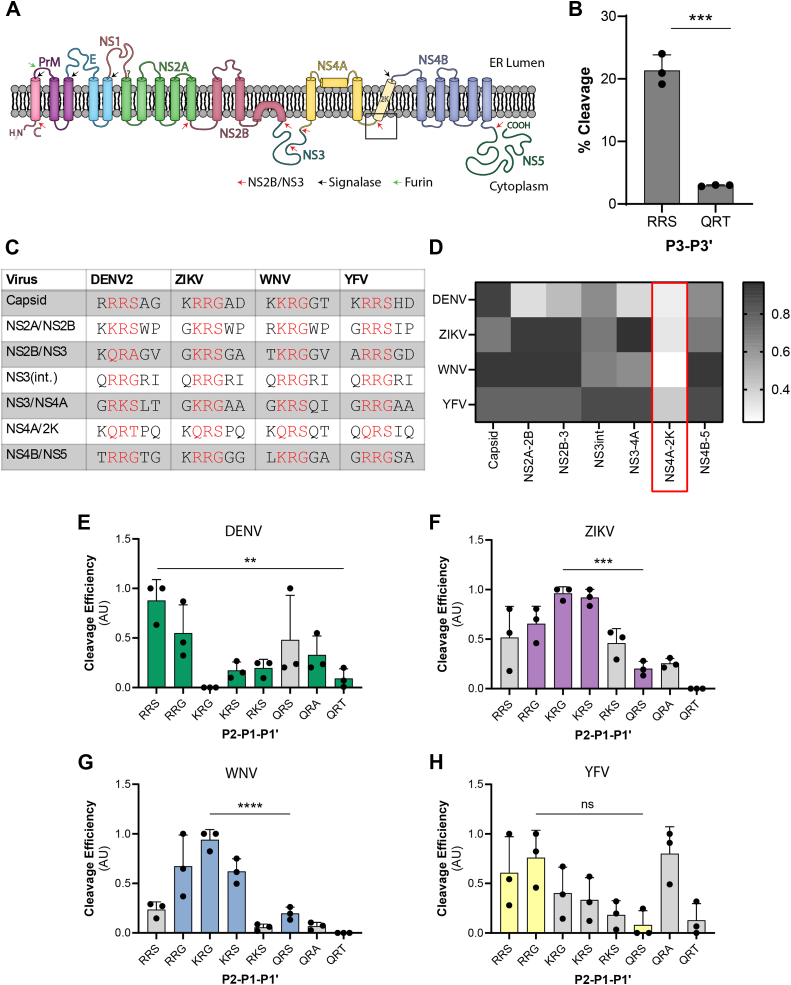
**Sequence-specific cleavage efficiency profiles**. *A*, model of a pre-processed flavivirus polyprotein translated into the membrane of the ER. *Red arrows* represent cytoplasmic cleavage junctions processed by the viral protease (NS2B/NS3pro). *Black arrows* indicate luminal cleavage junctions processed by host signalase. *Green arrow* represents the luminal cleavage junction processed by host furin. Cleavage junction between NS4A and the 2K peptide is marked by the *black box*. *B*, cleavage efficiency plot as determined by immunoblot defined as % cleavage of the capsid or NS4A|2K motif-encoding reporter. BHK-21 cells were lysed 24 h post co-transfection with the DENV protease and the indicated reporter construct. Bars represent the average cleavage efficiency of three experiments per condition and *black* circles indicate the cleavage efficiency calculated for each replicate. Statistical significance determined by unpaired *t* test. ∗∗∗*p* < 0.001. *C*, table representing the cleavage motifs at the seven cytoplasmic cleavage junctions processed by the viral proteases in the DENV2, ZIKV, WNV, and YFV polyproteins. Solved three amino acid cleavage motifs indicated in *red* letters. *D*, heat map of cleavage efficiencies at the indicated polyprotein cleavage junctions. NS4A|2K junction is marked by the *red box*. *E–H*, normalized cleavage efficiency plots as determined by immunoblot for eight indicated P2-P1′ sequences present in the cytoplasmic cleavage junctions of DENV (*D*), ZIKV (*E*), WNV (*F*), and YFV (*G*) polyproteins. U2OS cells were lysed 24 h post co-transfection with the indicated reporter and viral protease. Bars in color indicate the cleavage motifs present within the polyproteins that encode the tested protease. *Gray bars* represent sequences absent from the polyproteins of the indicated protease. Data are presented as efficiency of cleavage determined by intensity ratios in AU of the cleaved to total reporter signal relative to viral protease expression. Data for each independent experiment are normalized where the highest value is equal to one and the lowest value is equal to zero. Each bar represents the average cleavage efficiency of three experiments per condition and *black circles* indicate the cleavage efficiency calculated for each replicate. Statistical significance determined by one-way ANOVA followed by Tukey’s multiple comparisons *post hoc* test. ∗∗*p* < 0.01, ∗∗∗*p* < 0.001, ∗∗∗∗*p* < 0.0001, ns = not significant.

We next sought to explore the primary sequence of the substrate recognition motif as a molecular determinant for cleavage by orthoflavivirus proteases. It is known that the majority of the cytoplasmic sequences of the viral polyprotein cleavage motifs include two basic residues, typically arginines and/or lysines, followed by a small amino acid, typically serine or glycine (25, 28, 30, 56). However, when looking at the sequences present at the different cytoplasmic cleavage junctions within the polyproteins of several orthoflaviviruses, there is a notable degree of variance in the motifs that are processed at different sites within a single polyprotein, as well as differences in the sequences cleaved at the same site across different viral species (Fig. 5*C*). We specifically noticed the cleavage motif at the NS4A|2K junction of the DENV polyprotein differs from the previously identified consensus sequence with a glutamine at P2 and a threonine at P1’. To determine if there was a difference in cleavage efficiency between a consensus and non-consensus motif, we performed intracellular cleavage assays comparing reporters encoding the residues of the cytoplasmic capsid cleavage site (RRR|SAG) *versus* the NS4A|2K site (KQR|TPQ). We observed a significant decrease in cleavage efficiency of the NS4A|2K reporter compared to the reporter encoding the cytoplasmic capsid cleavage motif (Fig. 5*B*). Because of the dramatic difference between the two tested reporters, we next sought to investigate the degree of variability in the processing efficiency of the sequences located at all the cytoplasmic cleavage junctions of multiple orthoflavivirus polyproteins. We designed 8 reporter constructs encoding the non-repetitive protease recognition motifs (P2-P1′) within the cytoplasmic cleavage junctions of the DENV, ZIKV, WNV, and YFV polyproteins (Fig. 5, *A* and *C*). We then performed cleavage assays where one of the eight reporter constructs was co-expressed with a DENV, ZIKV, WNV, or YFV protease. Quantification of the immunoblots from these experiments revealed that each orthoflavivirus protease has distinct cleavage efficiencies for the different sequences present within their cytoplasmic polyprotein junctions and that these preferences varied across viral species (Fig. 5, *D*–*H*). For example, the ZIKV and WNV proteases had very similar graph patterns, while the DENV and WNV graph patterns were nearly opposite, similar to previous biochemical studies highlighting a difference in cleavage specificity between the two purified proteases (31). However, the most intriguing detail from this data is that the sequence within each viral polyprotein that was processed least efficiently by its protease was located at the same cytoplasmic cleavage junction: between NS4A and the 2K peptide (Fig. 5, *D*–*H*). We observed a statistically significant decrease in cleavage efficiency between the most efficiently processed reporters and those encoding the respective NS4A|2K motifs by the DENV, ZIKV, and WNV proteases (Fig. 5, *E*–*H*). This suggests that there is an essential role for the suboptimal cleavage of the motif at this position within the viral polyprotein. Together, these results revealed that each orthoflavivirus protease has a unique cleavage profile and suggests an evolutionary advantage to maintaining sequences of varying cleavage efficiencies at certain polyprotein cleavage sites.

### Cleavage of the NS4A|2K sequence is significantly delayed

The cleavage profiles provide insight into the viral proteases’ cleavage efficiency of different substrates after a 24-h post-transfection timepoint. However, we hypothesized that the timing of cleavage would be the most important factor in the context of polyprotein processing. Therefore, a more detailed way to study viral protease cleavage efficiency is to evaluate the cleavage kinetics of our reporters during infection. To investigate the intracellular cleavage kinetics, we can adapt our reporter system to long-term, time-lapse imaging of living cells (37). The visual advantage of our reporter construct is the nuclear translocation of the fluorescent reporter signal that occurs in response to cleavage of the protease recognition motif. Using this reporter system in combination with live-cell imaging allows us to study the efficiency of intracellular reporter cleavage over time by measuring the increase in reporter signal in the nucleus of the cell throughout viral infection (37).

**Figure 6:**
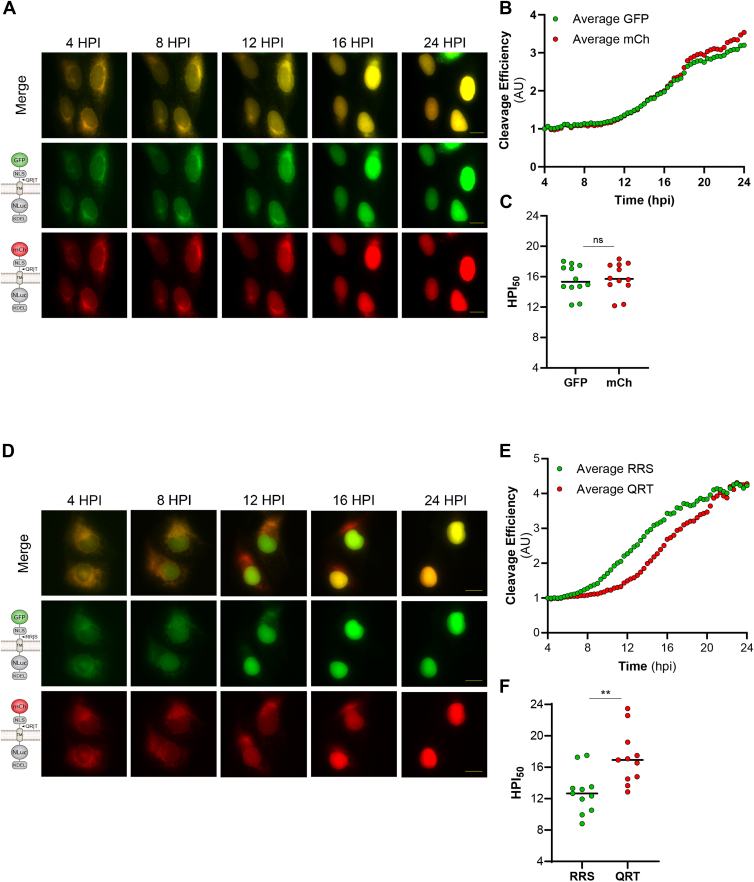
**Comparative cleavage kinetics of capsid *versus* NS4A|2K sequences.***A* and *D*, representative time-points of image series from live cell imaging of DENV-infected U2OS cells co-expressing (*A*) GFP-QR|T (*middle*) or (*D*) GFP-RR|S (*middle*) and mCh-QR|T (*bottom*) reporters. Merged images in *top panels*. Scale bars represent 20 μm. *B* and *E*, quantification of cleavage efficiencies of (*B*) GFP-QRT or (*E*) GFP-RRS *versus* mCh-QRT reporters, defined as the reporter signal intensity in the nucleus of infected cells co-expressing the two constructs (n = 12). Individual data points represent the average reporter intensity in the nucleus at the indicated time point. *C* and *F*, graphs representing the times at which the nucleus reaches the 50% maximum intensity (HPI50) of each reporter signal as determined by nonlinear regression analysis for each DENV-infected cell. Values for GFP *versus* mCherry reporters were compared using unpaired t-tests. ∗∗*p* < 0.01, ns = not significant.

To directly compare the intracellular cleavage kinetics of varying sequences, we designed reporters encoding different fluorescent proteins (GFP and mCherry) fused upstream of the two cleavage motifs. The fluorescent protein fused downstream of the TM domain in the original FlavER design was replaced with a NanoLuciferase to ensure that each reporter expresses a single fluorescent protein and that proper topology and localization of the reporter constructs are maintained. This method allows us to visualize and quantify the nuclear translocation of the two fluorescent signals that occur as a result of proteolytic cleavage of the respective reporter sequences within a single infected cell co-expressing the reporters. To utilize this assay, we first validated its efficacy by confirming that there was no inherent difference in the rate of nuclear translocation between GFP and mCherry. To confirm this, we designed two reporters encoding the same protease recognition sequence fused to different fluorescent proteins (GFP-QR|T, mCh-QR|T) and expressed them in cells together. The co-expressing cells were then infected with DENV 4 h before live-cell imaging for a 20-h timespan, where images were captured in 20-min intervals and compiled into movies. Using this method, we began to observe nuclear translocation of both GFP and mCherry at about 12 HPI, and the nuclear signal steadily increased as infection progressed. (Fig. 6*A* and Movie S1). Quantification of the signal intensities of both GFP and mCherry in the nucleus in each captured frame revealed no difference in the rate of nuclear translocation between the two fluorescent signals (Fig. 6*B*). We further calculated the average time at which the 50% maximum signal was reached in the nucleus (HPI50) during infection and observed no significant difference between GFP and mCherry (Fig. 6*C*). Together, these results indicate that this method can be used to compare the intracellular cleavage kinetics of two cleavage motifs fused to different fluorescent proteins during infection.

To further understand the significance of the variable cleavage sequences present in orthoflavivirus polyproteins, we aimed to directly compare the cleavage kinetics between the two protease recognition motifs found to be the most and least efficiently processed by the DENV protease. As shown in Figure 5, the DENV protease processed the RR|S sequence most efficiently and the QR|T sequence least efficiently. These motifs are located at the cytoplasmic capsid junction and NS4A|2K junction of the viral polyprotein, respectively. Therefore, we designed a reporter encoding a GFP upstream of the RR|S recognition motif (GFP-RR|S) to co-express with the mCh-QR|T reporter in cells to be infected with DENV (Fig. 6*D*). Using the same technique described previously, we found a distinct difference in the timing of the onset of nuclear translocation between the GFP and the mCherry signals (Movie S2). On average, we found that GFP began translocating at approximately 8 HPI, while mCherry did not begin translocating until approximately 12 HPI. Further, we observed complete nuclear translocation of GFP by ∼16 HPI and of mCherry by the endpoint of ∼24 HPI, on average (Fig. 6, *D* and *E*). Calculation of HPI50 during infection resulted in ∼13 HPI for GFP and ∼17 HPI for mCherry (Fig. 6*F*). Together, these results suggest a significant, approximately 4-h, delay in DENV protease cleavage of the QR|T sequence, located at the NS4A|2K junction within the viral polyprotein, compared to the RR|S motif.

### QR|T cleavage efficiency increases later in DENV infection

**Figure 7:**
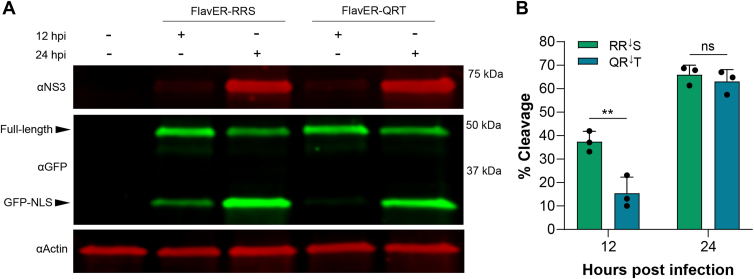
**Cleavage of capsid *versus* NS4A|2K sequences at 12 and 24 h post infection**. *A*, immunoblot for NS3, GFP, and actin of DENV-infected U2OS cells expressing GFP-RRS or GFP-QRT. Cells were infected with DENV at an MOI of 3 and lysed at either 12 or 24 h post infection. Labeled arrows denote the full-length and cleaved (GFP-NLS) reporter constructs. *B*, quantification of immunoblot defined as % cleavage of the reporter at 12 and 24 h post infection. Bars represent the average cleavage efficiency of three experiments per condition and *black* circles indicate the cleavage efficiency calculated for each replicate. Statistical significance determined by one-way ANOVA followed by Tukey’s multiple comparisons *post hoc* test. ∗∗*p* < 0.01, ns = not significant.

Next, we wanted to validate our cleavage kinetics results with our standard cleavage assays to highlight the importance of timing in the cleavage of intracellular sequences, particularly when considering polyprotein processing in the context of infection. To support the live-cell imaging results, we independently expressed GFP reporters encoding either the QR|T or the RR|S motifs in U2OS cells. These cells were then infected with DENV at an MOI of 3, and lysates were collected at 12 and 24 HPI. We observed that in our early infection time point, there is far more efficient cleavage of our RR|S reporter compared to the QR|T reporter, as indicated by the more robust cleavage band shown by immunoblot. However, by 24 HPI, we observed a strong cleavage band of our QR|T reporter that nearly mirrored the RR|S reporter cleavage efficiency (Fig. 7*A*). Signal intensity quantification of the intracellular cleavage assays paralleled these results. We observed a significant defect in QR|T reporter cleavage compared to that of the RR|S reporter motif at 12 HPI, yet there was no significant difference in cleavage efficiency between the two reporters by 24 HPI (Fig. 7*B*). These data accurately correspond with the trend observed in our live-cell cleavage kinetics assay using DENV infection and emphasizes the impact that progression of infection over time has on viral protease activity.

### Altering cleavage kinetics at the NS4A|2K junction is lethal

**Figure 8:**
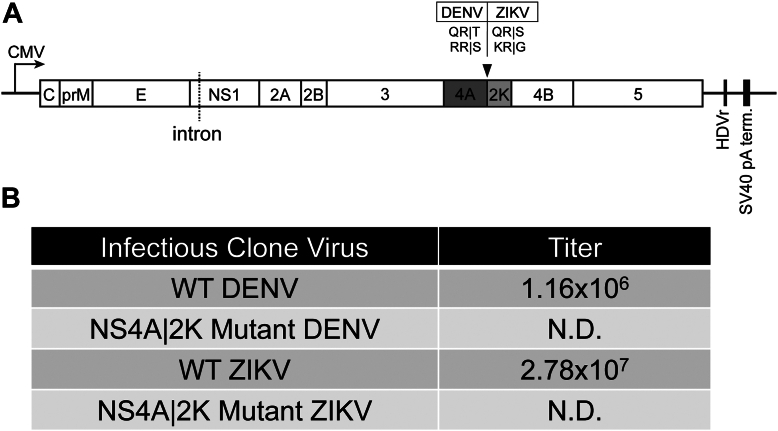
**Altering cleavage kinetics at the NS4A|2K junction is lethal for DENV and ZIKV.***A*, linear map of DNA-launched DENV and ZIKV infectious clones. Viral genomes lie between a CMV promotor and an HDV ribozyme (HDVr). An artificial intron is introduced in the NS1 gene segment to promote stability in bacteria. NS4A and 2K gene segments represented as *dark* and *light gray boxes*, respectively. *Black arrow* indicates the cleavage site where mutations were introduced. *B*, rescue of recombinant virus encoding the WT or fast cleavage sequence at the NS4A|2K junction within the polyproteins of the DENV or ZIKV infectious clones. N.D. = Not detectable.

We next wanted to understand the effect of polyprotein cleavage kinetics on the infectivity of orthoflaviviruses. To investigate this process, we utilized a DENV infectious clone that allows for the recovery of high viral titers after transfection into cells (57). We replaced the WT (QR|T) residues between NS4A and the 2K peptide with the cleavage motifs found to be the most efficiently processed by the DENV protease (RR|S) to increase the rate of cleavage at this junction within the viral polyprotein (Figs. 5*D* and 8*A*). We observed efficient recovery of WT virus, but we were unable to detect any foci from the cells incubated with the mutant supernatants (Fig. 8*B*). To see if this effect was conserved in another orthoflavivirus species, we utilized a ZIKV infectious clone (58). Using the same method described previously, we found that increasing the rate of cleavage at the NS4A|2K junction within the ZIKV polyprotein (QR|S → KR|G) also had lethal effects on viral recovery (Fig. 8*B*). These results suggest that suboptimal cleavage kinetics between NS4A and the 2K peptide within orthoflavivirus polyproteins are essential for viral fitness.

## Discussion

Orthoflavivirus proteases are essential protein complexes responsible for processing the viral polyprotein into its individual subunits that perform the functions to mediate infection. These proteases have also been shown to interact with different host proteins to establish a favorable environment for replication (59, 60). Because productive infections cannot be established without proper function of the viral protease, this protein complex has long been an attractive target for the development of antiviral therapeutics. However, no orthoflavivirus protease inhibitors have been successful in the clinic; thus, further understanding of its molecular functions is needed for the development of targeted antivirals (35). In this study, we sought to identify the subcellular determinants involved in orthoflavivirus protease-induced cleavage of substrates. We first used our tractable protease activity reporter system to identify two previously uncharacterized molecular factors associated with substrate cleavage by the DENV protease: membrane proximity of the recognition motif and ER subdomain localization of the substrate. We then investigated primary sequence specificity for multiple orthoflavivirus proteases as this was the only previously characterized cleavage determinant prior to this study. We found that although each orthoflavivirus protease had a unique cleavage profile, they all shared the characteristic of processing the sequence located at the NS4A|2K junction with the poorest efficiency. This led us to uncover the importance of the cleavage event for viral infectivity. Overall, this report investigated the molecular determinants for orthoflavivirus protease cleavage and further revealed that cleavage efficiency of the viral polyprotein plays a pivotal role in viral fitness.

Our FlavER reporter system is a valuable tool for studying intracellular cleavage specificity of orthoflavivirus proteases. Here, we show the reporter platform can accommodate various modifications to allow for the detailed investigation of substrate cleavage efficiency by viral proteases. First, we found that increasing the number of residues between the cleavage site and the TM domain in our reporter led to a correlative decrease in cleavage efficiency by the DENV protease (Fig. 2). This molecular determinant is consistent with what is proposed about the cytoplasmic cleavage junctions within the viral polyprotein, as the majority of the cleavage sites are assumed to be within less than 10 amino acids from the TM domain (Fig. 2*B*), which can be interpreted as being within close proximity to the ER membrane (61, 62, 63, 64). The cleavage sites presumed to be farther from the ER membrane, NS3int and NS3-NS4A, have both been shown to be strictly intramolecular cleavage events which could account for the tolerability of greater membrane distances (65). Further, several studies have revealed that cleavage of both the internal NS3 site and the NS3|NS4A junction is less efficient and kinetically slower than other cleavage events within the polyprotein which could be attributed to the membrane proximity of these protease recognition motifs (66, 67, 68). A limitation of the strategy to insert flexible linkers between the cleavage site and TM domain is the lack of structural data to definitively confirm the increased distance, which is technically challenging in the intracellular system employed in this study. Although insertion of flexible linkers is extensively used in molecular biology to increase the physical space between two domains, this does not exclude the possibility that the end-to-end distance between the separated motifs is unaffected. However, a previous structural study on the membrane-anchored tetraspanin-ADAM10 protease complex reported a distance-dependent effect on cleavage efficiency of membrane-bound substrates using similar linker extension strategies and cleavage assays (38). Further, our data support what is observed within the native viral polyprotein regarding the limited number of residues between the protease recognition motif of the cleavage junction and the nearest predicted TM domain (Fig. 2*B*).

We also found that the localization to specific subdomains of the ER had profound effects on the cleavage efficiency of the reporter constructs. We observed robust nuclear translocation of the fluorescent signals within the ER-sheet-localized reporters, indicating efficient cleavage by the DENV protease during infection (Fig. 4, *A* and *B*). In contrast, we observed that the viral protease is unable to cleave the reporter localized to ER tubules during DENV infection as evidenced by the complete lack of nuclear translocation of the reporter signal (Fig. 4*A*). This observation is consistent with reports that showed dsRNA, viral proteins, and assembled orthoflavivirus particles localized specifically in dilated ER cisternae (69, 70). It is possible that the lack of cleavage of Tubule-Rep could be attributed to its unique function of inducing positive curvature of the ER membrane; however, reticulophagy receptor 1 (RETREG1) is an ER-sheet localized host protein also known to induce membrane curvature that has been shown to be cleaved by several orthoflavivirus proteases (71, 72). Thus, the ability of the viral protease to cleave proteins encoding recognition motifs during infection is significantly impacted by their subcellular localization. However, there may be other determinants that regulate protease-mediated cleavage of proteins residing outside the ER cisternae that can be further investigated.

The final molecular determinant we investigated further was the primary sequence of the viral protease recognition motif. Many studies have reported on the cleavage specificity of orthoflavivirus proteases using purified recombinant proteases and *in vitro* enzymatic assays (31, 73, 74, 75, 76). Although these are valuable studies for gaining an understanding of protease activity, these methods take the viral protease out of the context of the cell and away from the membrane that it typically associates with. This limitation is eliminated with the use of our reporter system as we test the cleavage of intracellular substrates with a more native form of the viral protease that is exposed to natural cellular constraints. Using our reporter platform, we identified that the DENV, ZIKV, WNV, and YFV proteases each had distinct cleavage specificity patterns (Fig. 5). Understanding these specificity profiles has potential to provide insight into how cleavage efficiency at distinct cleavage sites affects the coordinated processing of the viral polyprotein. A few studies have determined that certain polyprotein cleavage events are prerequisites for processing at another site to occur; however, the chronological sequence of cleavage events of the orthoflavivirus polyproteins is largely unknown and warrants further investigation (22, 77, 78).

These results are also valuable for establishing parameters to use when evaluating potential host protein candidates that may be targeted for cleavage by orthoflavivirus proteases. Previous studies have uncovered three ER-resident host proteins known to be cleaved by multiple orthoflavivirus proteases: stimulator of interferon genes (STING), RETREG1, and diacylglycerol O-acyltransferase 2 (DGAT2). Both STING and RETREG1 are known to restrict orthoflavivirus infection (71, 79, 80, 81). In response, multiple orthoflavivirus proteases evolved the ability to cleave these host proteins at non-consensus cleavage sequences in order to inhibit their antiviral functions (71, 82, 83, 84). DGAT2 is unique from STING and RETREG1 in that cleavage by the viral protease stabilizes this host protein for the virus to hijack its function as a proviral factor (85). DGAT2 is known to promote the formation of lipid droplets which have been shown to serve as an energy source of viral replication (86, 87, 88). Thus, enhancing the stability of DGAT2 allows for lipid droplet accumulation that creates a favorable environment for viral replication. Other non-ER-resident host proteins, specifically factors localized to the mitochondria, have also been shown to be cleaved by orthoflavivirus proteases (89, 90). Our results would suggest that these proteins are likely interacting with other membrane proximal host or viral proteins that allow the protease to access the cleavage site. Identifying host proteins that are cleaved by orthoflavivirus proteases is a promising area of research as it provides an understanding of how this viral protein complex interacts with cellular factors to promote replication. The results of this study can support these research efforts by identifying important parameters to acknowledge when evaluating cleavage targets. Considering the ER subdomain localization of the host protein and the primary sequence and membrane proximity of the potential cleavage site could allow for a more accurate assessment of its cleavage probability.

When investigating primary sequence specificity, we also identified that each protease processed the sequence located at the NS4A|2K junction least efficiently and that proteolytic processing of the NS4A|2K (QR|T) sequence was significantly delayed compared to the capsid cleavage sequence (RR|S) during DENV infection (Figs. 5, and 6, *D*–*F*). Notably, we observed that in contrast to the cleavage assays with exogenous protease expression, the degree of QR|T cleavage nearly matches that of RR|S at 24 h in the context of DENV infection (Fig. 7). These results indicate that cleavage of the NS4A|2K sequence may require a specific environment consisting of a high concentration of protease and/or infection-induced membrane rearrangements, which cannot be simulated in protease transfections. Interestingly, ultrastructural studies of infected cells have indicated these viruses induce temporal changes to the ER membrane that begin with the formation of membrane packets that go on to enclose *de novo* vesicles as infection progresses (91). Thus, it is possible that efficiency-regulated cleavage of the viral polyprotein is linked to transitional virus-induced membrane alterations.

Lastly, we sought to determine if the delayed cleavage event at the NS4A|2K junction of the orthoflavivirus polyprotein was critical for viral fitness and found that introducing a high efficiency cleavage site between NS4A and the 2K peptide led to complete loss of infectious viral progeny (Fig. 8). These results suggest that there is an essential purpose for the delay in proteolytic processing at this particular cleavage site. One possibility for this function is that a cleavage delay is required for efficient replication to occur during early infection. This hypothesis is supported by recent studies that have shown that the unprocessed NS4A-2K-NS4B precursor operates as a functional intermediate that interacts with other viral proteins. Specifically, two separate reports found that the NS4A-2K-NS4B intermediate interacts with both NS1 and NS3 and that these interactions are required for DENV replication (92, 93, 94). These studies bolster the idea that the purpose behind the delay in NS4A|2K processing is due to the functions performed by NS4A-2K-NS4B in its pre-cleaved form. This could suggest that premature separation of NS4A and NS4B due to the introduction of a more efficient cleavage sequence may interrupt these crucial interactions and inhibit replication resulting in the loss of fitness (Fig. 7). Future studies are targeted at further understanding the impact of temporal polyprotein processing on virus infection.

Collectively, this report identified membrane proximity and ER subdomain localization as molecular determinants associated with substrate cleavage by orthoflavivirus proteases and highlighted the impact of sequence specificity on polyprotein processing and subsequent viral fitness. Our data further provides a set of parameters to consider when evaluating promising host protein candidates that may be targeted by these viral proteases. Together, these results advance our understanding of the intracellular molecular activities of orthoflavivirus proteases which can contribute to closing the gap in knowledge of their complex mechanisms to support the development of targeted antiviral therapeutics.

## Experimental procedures

### Cells and viruses

Human embryonic kidney (HEK)-293T cells (ATCC, CRL-3216) and U2OS osteosarcoma cells (ATCC, HTB-96) were cultured in Dulbecco’s modified Eagle’s medium (DMEM) supplemented with 10% fetal bovine serum (FBS) and 100 U/ml penicillin/streptomycin (P/S). Vero E6 cells (a gift from Kevin Harrod, University of Alabama at Birmingham) were cultured in modified Eagle’s medium (MEM) supplemented with 10% FBS and 100 U/ml P/S. C6/36 *Aedes albopictus* cells (ATCC, CRL-1660) were cultured in DMEM supplemented with 10% FBS and 100 U/ml P/S. All mammalian cells were maintained in a humidified environment at 37 °C. C6/36 cells were maintained in a humidified environment at 28 °C. All cells were routinely tested for *mycoplasma* using MycoStrip *Mycoplasma* Detection Kit (Invivogen).

DENV serotype 2 strain 16681 and ZIKV-MR766 were rescued from HEK-293T cells transfected with pcDNA6.2 DENV2 16681 and pcDNA6.2 ZIKV MR766 plasmids, respectively, as described previously (57, 58). Supernatants were then passaged over C6/36 cells to propagate the virus. Supernatants were collected and clarified to remove cell debris by centrifugation at 2300×*g* for 15 min at 4 °C. Clarified supernatants were aliquoted and stored at −80 °C.

### Plasmid construction

The WT FlavER construct was generated as previously described (37).

#### Membrane proximity reporters

Cloning of the FlavER reporters with extended flexible (glycine-glycine-serine) linker regions was achieved through site-directed mutagenesis. Site-specific forward and reverse primers were used with T7_F (5′-TAATACGACTCACTATAGGG-3′) and FlavER-StuI_R (5′-CGGGAGCTTTTTGCAAAAGCC-3′) primers to generate overlapping PCR fragments that were ligated into the FlavER vector linearized with NheI and StuI *via* HiFi assembly (NEB). Site-specific primer pair sequences are shown in Table 1. Ligations were transformed into DH5α *E. coli* (Zymo Research).

**Table 1:** Primer sequences to insert linkers into FlavER

Primer Name	Sequence (5′-3′)
FlavER-8aa_F	GAACGGATCCGGCTATGGGACTATTGCTGTGATC
FlavER-8aa_R	CCCATAGCCGGATCCGTTCAGTCCAGCTGACCTCC
FlavER-11aa_F	GAACGGATCCGGAGGCTCTGGCTATGGGACTATTGCTGTGATC
FlavER-11aa_R	CCATAGCCAGAGCCTCCGGATCCGTTCAGTCCAGCTGACCTCC
FlavER-14aa_F	CTGAACGGATCCGGAGGCTCTGGTGGAAGCGGCTATGGGACTATTGCTGTGATC
FlavER-14aa_R	CCCATAGCCGCTTCCACCAGAGCCTCCGGATCCGTTCAGTCCAGCTGACCTCC
FlavER-17aa_F	GAACGGATCCGGAGGCTCTGGTGGAAGCGGTGGCTCCGGCTATGGGACTATTGCTGTGATC
FlavER-17aa_R	CATAGCCGGAGCCACCGCTTCCACCAGAGCCTCCGGATCCGTTCAGTCCAGCTGACCTCC

#### ER subdomain reporters

Tubule-Rep was constructed through amplification of *RTN4A* (a gift from Carolyn Coyne, Duke University), using Rtn4a-Rep_F and Rtn4a-Rep_R (Table 2). The protease recognition motif, NLS, and GFP were amplified from FlavER using Rtn4a-RRS_F and GFP-NLS_R. The two fragments were HiFi assembled into the FlavER backbone digested with NheI and ApaI. Sheet-Rep was constructed through amplification of *SIGMAR1* from the cDNA of HEK-293T cells using SigmaR1-Rep_F and SigmaR1-Rep_R. This product was HiFi assembled with FlavER digested with BamHI and ApaI. Ligations were transformed into DH5α *E. coli.*

**Table 2:** Primers sequences for ER-subdomain reporters

Primer Name	Sequence (5′-3′)
Rtn4a-Rep_F	CAAGCTGGCTAGCCACCATGGAAGACCTGGACCAGTC
Rtn4a-Rep_R	CTCCTTTTTGCAGCGGTACCCCGTTCATAAATAACAGGAACAC
Rtn4a-RRS_F	CAAAAAGGAGGTCAGCTGGACTGAACGGATCCATGGTGAGCAAGGGCGAGG
GFP-NLS_R	GATCAGCGGGTTTAAACGGGCCCTCTAGATCAAACTTTTCTTTTTTTTTTAG
SigmaR1-Rep_F	GGAGGTCAGCTGGACTGAACGGATCCTGGGCGTGGGCCGCGCTGCTC
SigmaR1-Rep_R	GATCAGCGGGTTTAAACGGGCCCTCATAGCTCGTCTTTAGGGTCCTGGCCAAAGAGG

#### Polyprotein cleavage motif reporters

Cloning of the FlavER reporters with varying cleavage site sequences was accomplished through standard restriction enzyme cloning. Sequence-specific oligos were annealed and ligated into the KpnI and BamHI linearized FlavER vector using T4 DNA ligase (NEB) for 30 min at room temperature (RT). Annealed oligo sequence pairs are shown in Table 3. Ligations were transformed into DH5α *E. coli*.

**Table 3:** Primers for site-directed mutagenesis of cleavage motif

Primer Name	Sequence (5′-3′)
FlavER-RRS_F	CGCTGCAAAAAGGAGGTCAGCTGGACTGAACG
FlavER-RRS_R	GATCCGTTCAGTCCAGCTGACCTCCTTTTTGCAGCGGTAC
FlavER-RRG_F	CGCTGCAAAAAGGAGGGGCGCTGGACTGAACG
FlavER-RRG_R	GATCCGTTCAGTCCAGCGCCCCTCCTTTTTGCAGCGGTAC
FlavER-KRG_F	CGCTGCAAAAAAGAGGGGCGCTGGACTGAACG
FlavER-KRG_R	GATCCGTTCAGTCCAGCGCCCCTCTTTTTTGCAGCGGTAC
FlavER-KRS_F	CGCTGCAAAAAAGAGGTCAGCTGGACTGAACG
FlavER-KRS_R	GATCCGTTCAGTCCAGCTGACCTCTTTTTTGCAGCGGTAC
FlavER-RKS_F	CGCTGCAAAAAGGAAGTCAGCTGGACTGAACG
FlavER-RKS_F	GATCCGTTCAGTCCAGCTGACTTCCTTTTTGCAGCGGTAC
FlavER-QRS_F	CGCTGCAAAACAGAGGTCAGCTGGACTGAACG
FlavER-QRS_R	GATCCGTTCAGTCCAGCTGACCTCTGTTTTGCAGCGGTAC
FlavER-QRA_F	CGCTGCAAAACAGAGGGCCGCTGGACTGAACG
FlavER-QRA_R	GATCCGTTCAGTCCAGCGGCCCTCTGTTTTGCAGCGGTAC
FlavER-QRT_F	CGCTGCAAAACAGAGGACTGCTGGACTGAACG
FlavER-QRT_R	GATCCGTTCAGTCCAGCAGTCCTCTGTTTTGCAGCGGTAC

#### Live-cell imaging reporters

Generation of the modified reporters used for live-cell imaging was performed using standard restriction cloning. The mCherry was removed from the FlavER-RR|S and QR|T vectors through digestion with EcoRI and ApaI and replaced with a NanoLuciferase that was synthesized as a gBlock with homology to the reporter vector and ligated using T4 DNA ligase for 30 min at RT. The GFP from GFP-QR|T-NLuc was cut out of the vector using NheI and XhoI and replaced with the mCherry digested from pcDNA3.1(+)-mCherry using the same restriction enzymes. The two products were ligated using T4 DNA ligase for 30 min at RT. Ligations were transformed into Stable Competent *E. coli* (NEB).

#### Viral proteases

The construction of expression plasmids encoding DENV NS2B-3-V5, DENV NS2B-3-S135A-V5, ZIKV NS2B-3-V5, WNV NS2B-3-V5, and YFV NS2B-3-V5 have been described previously (37, 71).

#### Site-directed mutagenesis of infectious clones

Mutations to the NS4A|2K junctions of pcDNA6.2 DENV2 16681 and pcDNA6.2 ZIKV MR766 plasmids were introduced through site-directed mutagenesis. Site-specific forward and reverse primers were used with DENV2-NS3-XhoI_F (5′-GTATAGCAGCTAGAGGATACATCTC-3′) and DENV2-NS5-StuI_R (5′-CTTCGTGTCCTGGTCCTCCTTTTG-3′) primers for pcDNA6.2 DENV2 16681 and ZIKV-NS3-SbfI_F (5′-AGCAGTTGCTCTGGACTACCC-3′) and ZIKV-NS5-MluI_R (5′-GATAAACTCTTCTTTGGTGCAG-3′) primers for pcDNA6.2 ZIKV MR766 to generate overlapping PCR fragments that were ligated into the pcDNA6.2 DENV2 16681 or pcDNA6.2 ZIKV MR766 backbone linearized with XhoI and StuI or SbfI and MluI, respectively, *via* HiFi assembly (NEB). Site-specific primer pair sequences are shown in Table 4. Ligations were transformed into Stbl2 Competent *E. coli* (Thermofisher).

**Table 4:** Site-directed mutagenesis primers for viral genomes

Primer Name	Sequence (5′-3′)
DENV2-4A|2K-RRS_F	AGACGCAGATCTGCAGGCGACAACCAACTGACCTACGTTG
DENV2-4A|2K-RRS_R	GCCTGCAGATCTGCGTCTTTCAGGTTCTGGAATAAGCAAAAC
ZIKV-4A|2K-KRG_F	CCCGAGCCAGAGAAGAAAAGAGGGCCCCAAGATAACCAGATGG
ZIKV-4A|2K-KRG_R	CTGGTTATCTTGGGGCCCTCTTTTCTTCTCTGGCTCGGGTATG

### Molecular modeling

The three-dimensional structure of the NS2B-NS3-NS4A polyprotein (NS^2B-4A^) was generated using Alphafold3 (95). Specifically, we modeled residues 1341 to 2220 to ensure that NS3 was adequately tethered to the membrane by NS2B and NS4A of the polyprotein. This structure was then modeled into a POPC membrane using VMD (Visual Molecular Dynamics) program (96). Briefly, NS^2B-4A^ was solvated using Helmut Gubmuller’s SOLVATE program (https://www.mpinat.mpg.de/grubmueller/solvate). The POPC membrane and NS^2B-4A^ were then aligned using their center of mass and NS^2B-4A^ was then incorporated into the membrane. The entire system was then solvated and neutralized by adding the appropriate number of NA ^+^ ions using VMD. We then used NAMD to perform molecular dynamics (MD) simulations to ensure the model generated was appropriately equilibrated and minimized (97). We used CHARMM36 force-field parameters for all steps of this process (98). Briefly, we first performed minimization and equilibration on the system with all atoms constrained except the lipid tails to appropriately relax the lipid bilayer. We then repeated minimization and equilibration with only the protein constrained to appropriately relax the environment. Finally, the entire system was equilibrated using a short simulation (0.5 ns) at constant temperature (300K) and pressure (1 atm).

### Lentivirus production and transduction

The production of lentiviral vectors expressing the indicated reporter transgenes was performed using the same method as described previously (37). Briefly, (1.5 × 10^6^) HEK-293T cells were transfected with 1 μg pLJMI-reporter, 0.75 μg psPAX2 (Addgene plasmid #12260), and 0.25 μg pCAGGS-VSV G-Kan using polyethyleneimine (PEI). 48 h post-transfection, lentivirus was harvested by passing cell supernatants through 0.2-micron filters (Thermo Scientific, 725–2520). U2OS cells (5 × 10^5^) were transduced with 500 μL of each lentiviral vector stock for 48 h prior to re-seeding into an 8-well chamber or live-imaging slides.

### Immunofluorescence staining/microscopy

U2OS cells (40,000 cells/well) stably expressing reporter constructs were infected with DENV or reverse transfected with the indicated V5-tagged DENV protease in an 8-well chamber slide (Celltreat). After 24 h, cells were fixed in 4% paraformaldehyde in phosphate-buffered saline (PBS), permeabilized with 0.1% Triton X-100 (Fisher) diluted in PBS, and washed with PBS followed by incubation with mouse α-V5 epitope tag monoclonal antibody (Invitrogen, 46–0705) or recombinant α-double-stranded RNA (dsRNA) monoclonal antibody (Kerafast) for 1 h at RT. The cells were then washed with PBS 3X for 5 min each and probed for 30 min with goat α-mouse conjugated to Alexa Fluor-647 (Invitrogen, A21236) at RT. Following secondary antibody incubation, cells underwent 3 PBS washes for 5 min each prior to incubation in PBS containing 300 nM DAPI for 5 min at RT. The slide was mounted using ProLong Diamond Antifade Mountant (Invitrogen) and a 24 × 50 mm Premium Superslip (FisherScientific). Cells were imaged on the 60X objective of an Olympus IX83 inverted fluorescent microscope.

### Intracellular cleavage assay

Protease overexpression: U2OS or BHK-21 cells (200,000 cells/well) were co-transfected with the indicated reporter and protease plasmids using PEI at a 1:1 ratio of DNA (μg) to 1 mg/ml PEI stock (μL). 24 h post transfection, cells were lysed in 1× RIPA + protease inhibitor cocktail (Sigma).

DENV Infection: U2OS cells (100,000 cells/well) were transduced with the indicated reporter lentiviral vectors 48 h prior to infection with DENV at an MOI of 3. Cells were lysed in 1× RIPA + protease inhibitor cocktail (Sigma) at 12 and 24 h post-infection.

Lysates were clarified by centrifugation at 12,000×*g* for 10 min prior to separation by SDS-PAGE using a 4 to 20% Tris-glycine polyacrylamide pre-cast gel (BioRad) and transfer to nitrocellulose membranes. After 30 min of blocking in PBS + 10% non-fat milk, membranes were probed using the indicated primary antibodies: mouse α-V5 (Invitrogen), rabbit α-GFP (Proteintech), mouse α-GFP (Proteintech), mouse α-actin (Proteintech), and/or rabbit α-DENV NS3 (GeneTex), followed by incubation with corresponding near-infrared dye-conjugated secondary antibodies (LiCor) diluted in PBST + 5% non-fat milk and imaged on an Odyssey CLx imaging system (LiCor).

### Long-term fluorescent live-cell imaging

3 × 10^4^ U2OS cells co-expressing two reporter constructs (GFP-QRT and mCherry-QRT or GFP-RRS and mCherry-QRT) were seeded in an 8-well live-imaging slide (Ibidi). The following day, cells were infected with DENV at an MOI of 10. Infection was synchronized at 4 °C for 1 h, followed by shifting cells to 37 °C for 3 h. 4 h post-infection, the imaging slide was transferred to a CO2-controlled incubated chamber positioned over a motorized inverted microscope. Images were taken every 20 min for a 20-h period, ending at 24 HPI. Image series were exported and compiled using ImageJ software (NIH), and multi-panel images were cropped and rendered in Photoshop CC 2021 (Adobe).

### Focus forming assay

HEK-293T cells were transfected with the WT or mutated DENV or ZIKV infectious clone plasmids. 72 h post transfection, supernatants were collected and serially diluted over Vero E6 cells for 48 h. Cells were fixed in 4% paraformaldehyde (Electron Microscopy Sciences) diluted in PBS for 10 min at RT. Cells were then permeabilized with PBS + 0.1% Triton 100-X for 10 min at RT followed by a 5-min wash with PBS. Monolayers were incubated with α-orthoflavivirus E-protein monoclonal antibody (clone 4G2, ATCC VR-1852) overnight at 4C. Cells were then washed with PBS followed by incubation with α-mouse antibody conjugated to Alexa Fluor 488 (Invitrogen A11029) for 30 min at RT. Foci were counted using an Olympus IX83 inverted fluorescent microscope.

### Image and data analysis

Models were generated using Photoshop, Adobe Illustrator, and Biorender. Immunoblots were uploaded and quantified in ImageStudio. Quantitative values were determined by dividing the cleavage band by the total reporter signal, followed by normalization to protease expression. Data was plotted in Prism 9 Software (Graphpad).

Intensity plot profile analysis was performed using the Plot Profile tool in ImageJ. Data was plotted in Prism 9 Software (Graphpad).

ImageJ was used to quantify fluorescent signal intensity in the nucleus of cells from live-cell time-lapse images. Data was plotted in Prism 9 Software (Graphpad).

### Statistics

Non-linear regression analysis, unpaired t-tests, and one-way ANOVA were performed in Prism 9 Software (Graphpad).

## Data availability

All the data generated or analyzed during the study are included in the manuscript.

## Supporting information

This article contains supporting information.

## Conflict of interest

The authors declare that they have no conflicts of interest with the contents of this article.
