# Supplementary material for: Subcellular determinants of orthoflavivirus protease activity
Source: J Biol Chem. 2025 Jul 5;301(8):110451. doi: 10.1016/j.jbc.2025.110451 (PMC12336820; doi:10.1016/j.jbc.2025.110451)
Supplement: Supporting Information [file mmc3.docx]

**Supporting Information**

**Supplemental Movie 1: Nuclear translocation kinetics of GFP versus mCherry**

Live-cell imaging of DENV-infected cells co-expressing GFP-QR|T and mCh-QR|T reporters. Imaging began 4 hours post infection with images captured every 20 minutes for a 20-hour time span.

**Supplemental Movie 2: Cleavage kinetics of GFP-RR|S versus mCh-QR|T**

Live-cell imaging of DENV-infected cells co-expressing GFP-RR|S and mCh-QR|T reporters. Imaging began 4 hours post infection with images captured every 20 minutes for a 20-hour time span.
